# Dynamic Changes in Amygdala Psychophysiological Connectivity Reveal Distinct Neural Networks for Facial Expressions of Basic Emotions

**DOI:** 10.1038/srep45260

**Published:** 2017-03-27

**Authors:** Matteo Diano, Marco Tamietto, Alessia Celeghin, Lawrence Weiskrantz, Mona-Karina Tatu, Arianna Bagnis, Sergio Duca, Giuliano Geminiani, Franco Cauda, Tommaso Costa

**Affiliations:** 1 Department of Medical and Clinical Psychology, and CoRPS - Center of Research on Psychology in Somatic diseases - Tilburg University, Tilburg, 5000LE, The Netherlands; 2 Department of Psychology, University of Torino, via Po 14, Torino, 10123, Italy; 3 Department of Experimental Psychology, University of Oxford, 9 South Parks Road, Oxford, OX1 3UD, United Kingdom; 4 GCS-fMRI, Kolliker Hospital, Torino, 1100, Italy

## Abstract

The quest to characterize the neural signature distinctive of different basic emotions has recently come under renewed scrutiny. Here we investigated whether facial expressions of different basic emotions modulate the functional connectivity of the amygdala with the rest of the brain. To this end, we presented seventeen healthy participants (8 females) with facial expressions of anger, disgust, fear, happiness, sadness and emotional neutrality and analyzed amygdala’s psychophysiological interaction (PPI). In fact, PPI can reveal how inter-regional amygdala communications change dynamically depending on perception of various emotional expressions to recruit different brain networks, compared to the functional interactions it entertains during perception of neutral expressions. We found that for each emotion the amygdala recruited a distinctive and spatially distributed set of structures to interact with. These changes in amygdala connectional patters characterize the dynamic signature prototypical of individual emotion processing, and seemingly represent a neural mechanism that serves to implement the distinctive influence that each emotion exerts on perceptual, cognitive, and motor responses. Besides these differences, all emotions enhanced amygdala functional integration with premotor cortices compared to neutral faces. The present findings thus concur to reconceptualise the structure-function relation between brain-emotion from the traditional one-to-one mapping toward a network-based and dynamic perspective.

Affective neurosciences are concerned with understanding how emotions are represented in brain activity and how this translates into behaviour, thereby characterizing structure-to-function relations. Traditionally, lesion studies informed early localization approaches by outlining the deficits in emotion processing that follow focal brain damage^1^. Although lesion studies and neuropsychological observations remain fundamental for inferring structure-function relations and translating correlational observations to causation, functional neuroimaging has become the primary method for characterizing the brain basis of emotions in the past two decades^2^.

These functional magnetic resonance imaging (fMRI) investigations are improving our understanding of which individual brain regions respond to specific emotions. Moreover, the sizable fMRI literature accumulating in recent years has enabled quantitative meta-analyses to assess the consistency and specificity of the neural signature of emotion processing beyond idiosyncrasies of individual studies. Yet results are mixed, with some original studies and meta-analyses supporting the existence of discrete and non-overlapping neural correlates for different emotions^3^,^4^,^5^,^6^,^7^,^8^, and others reporting little evidence that discrete emotion categories can be localized in distinct and affect-specific brain networks^2^,^9^,^10^.

Common to most of these approaches scrutinizing the neural signatures of basic emotions is the reliance on methods that characterize the static response of individual areas, rather than their dynamic connectional changes. This focus on ‘disconnected’ brain structures, however, can be incomplete in representing how complex functions like emotion processing map into brain activity. In fact, the role of a given structure is partly determined by the dynamic interactions it entertains with other regions, thus shifting the focus toward inter-regional connectivity patterns^11^,^12^. Addressing the issue of how emotions are embedded in dynamic neural networks distributed across large spatial scales thus promises to re-conceptualize the longstanding debate on the neural bases of emotions according to a more neurobiologically plausible and contemporary view of the structure-function relation^13^,^14^,^15^. A given area can be involved in processing different emotions rather than only one single emotion^16^. Nevertheless, the critical neural signature that differentiates one specific emotion from the others can be found in the unique pattern of inter-regional connectivity and synchrony amidst areas. Additionally, emotion processing is a multi-componential phenomenon that entails different functions, from stimulus recognition to subjective experience or feelings, from the enactment of expressive and instrumental behaviours to memory formation^17^,^18^. Nevertheless, the bulk of the literature examining the neural correlates of emotion processing concentrated primarily on visual perception of facial expressions^19^,^20^, akin to the original studies addressing the existence of discrete basic emotions, which tested recognition of prototypical facial expressions^21^. Faces are indeed one of the most powerful and richest tools in the communication of social and affective signals^22^ and, besides their affective value, emotionally neutral faces elicit consistent response in emotion-sensitive structures, including the amygdala^23^,^24^.

The amygdala is indeed a key structure in the perception and response to emotional signals and has been classically linked to fear processing^25^,^26^,^27^,^28^. However, more recent findings have extended its functions to recognition of other emotions^29^,^30^ or to multiple processes beyond emotion perception, including memory formation, reward processing or social cognition^31^,^32^,^33^. For example, a recent activation likelihood estimation (ALE) meta-analysis reported that five out of the seven basic emotions (anger, disgust, fear, happiness and sadness) demonstrated consistent activation within the amygdala^3^. Amygdala voxels were found to contribute to the classification of happiness and disgust, in addition to fear, when multivariate pattern analysis (MVPA) techniques were applied to decode brain activity patterns induced by exposure to short movies or mental imagery^6^. Lastly, lesion studies in patients with selective bilateral amygdala damage reported that some of these patients may still be able to recognize fear from different stimuli, thus showing that fear perception does not invariably depend on the necessary contribution of the amygdala^34^,^35^,^36^,^37^.

Although this heterogeneity of functions in the amygdala has been initially taken as suggesting that emotions do not have a characteristic or unique neural signature^2^, these findings can more parsimoniously indicate that a one-to-one mapping between single instances of emotions and brain regions is too simplistic^11^. Current conceptions emphasize indeed how this functional diversity parallels the intricacy of amygdala circuitry and long-range connections, underscoring that brain regions do not have functions in isolation, but can fulfil multiple functions depending on the networks they belong to^31^,^32^,^33^. Understanding these context- and emotion-dependent changes of amygdala inter-regional connectional patterns is thus of crucial importance given its implication in virtually all psychiatric or neurological states characterized by social deficits, including addiction, autism or anxiety disorders^38^,^39^.

A host of techniques can be used to examine how spatially remote brain regions interact during emotion processing by gauging brain activity across time. One of the most popular and successful approaches for examining dynamic and task-related interactions between one region and the rest of the brain is to assess psychophysiological interactions (PPI)^40^,^41^. PPI analyses whether an experimental manipulation (e.g., exposure to an emotional stimulus) changes the coupling between a source region and other brain areas. Prior studies have investigated with PPI how exposure to one type of emotional stimuli (e.g., fearful faces) alters amygdala connectivity compared to face perception^42^, or context-based interregional covariance of amygdala response across broadly different tasks and domains such as emotion perception, attention, decision-making or face perception^43^,^44^. To our knowledge, however, the emotion-specific connectional fingerprint of human amygdala and its possible dynamic changes as a function of exposure to different facial expressions has not yet been investigated. To this end, we applied PPI analysis to examine whether the perception of angry, disgusted, fearful, happy and sad facial expressions significantly modulates amygdala co-activation with the rest of the brain compared to neutral faces, and whether these dynamic and emotion-specific changes in amygdala’s connectivity profile characterize distinctively the response to discrete emotional categories. Our focus on perception of facial expressions enabled a closer comparison with previous neuroimaging studies that also studied visual perception of the same emotional signals, albeit with different fMRI methods, and warranted robust signal change in the amygdala, which is a pre-requisite for the successful application of PPI analysis.

## Results

**Figure 1:**
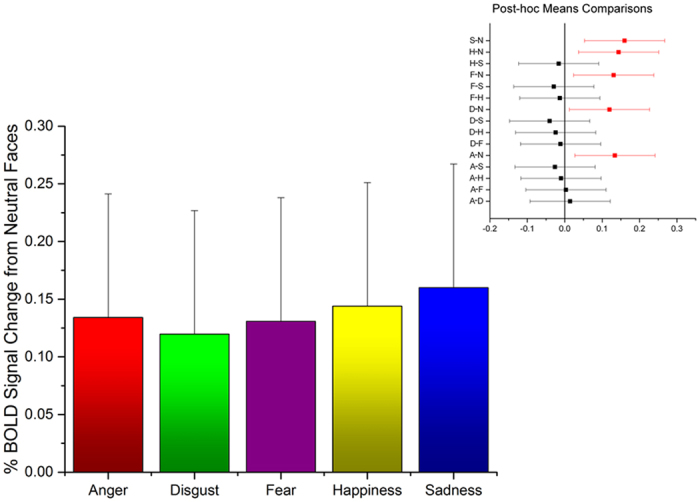
Difference in amygdala percentage of BOLD signal change between each emotional expression and neutral faces (mean ± SEM). The inset reports the results of post-hoc comparisons between each possible pair of expressions (mean ± SEM; red lines denote statistically significant differences at *p* < 0.05). *Abbreviations*: A, Anger; D, Disgust; F, Fear; H, Happiness; N, Neutral; S, Sadness.

A preliminary analysis assessed the percentage of blood oxygen level dependent (BOLD) signal change in the amygdala in response to the different stimulation conditions. Figure 1 reports the difference in amygdala fMRI response between each emotional expression and neutral faces. The fMRI data were entered in a repeated-measures Analysis of Variance (ANOVA) with the six-levels within-subjects factor ‘Facial Expressions’ (angry, disgusted, fearful, happy, sad, and neutral facial expressions). The difference in the mean fMRI signal change evoked in the amygdala by the six expressions was statistically significant [*F*(5, 80) = 3.05, *p* = 0.001]. Post-hoc comparisons revealed that amygdala activity increased significantly in response to all 5 emotional expressions, as compared to the neutral expression (*p* ≤ 0.012, by Fisher LSD test), whereas there was no difference in amygdala response amidst emotions (*p* ≥ 0.39).

**Figure 2:**
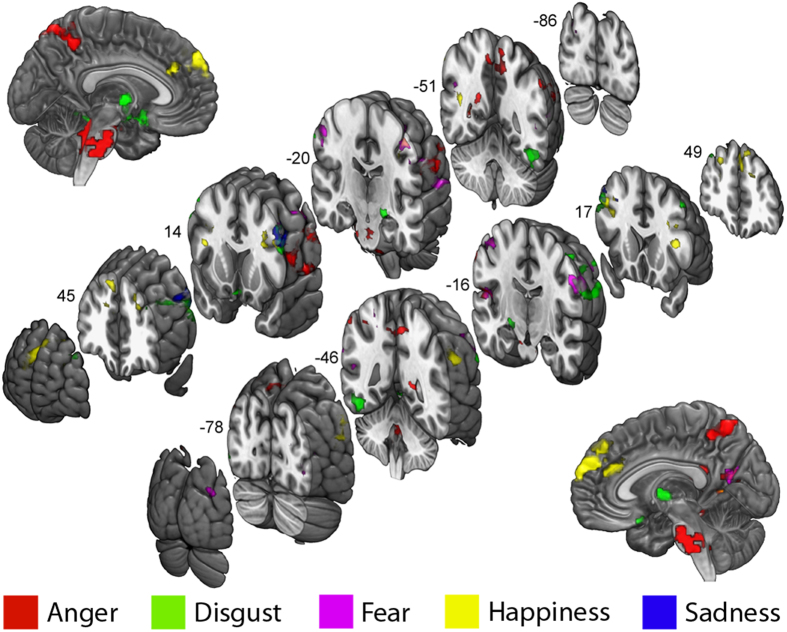
Brain areas showing significant emotion-dependent functional interactions with the amygdala for the contrast of each emotion with the neutral expression condition separately. MNI *Y* coordinates are reported for each slice. Results are whole-brain thresholded at *Z* > 2 and cluster corrected at *p* < 0.05.

The PPI analysis of fMRI data revealed that neural activity in multiple cortical and subcortical areas co-varied, and was therefore functionally correlated, with the activity in the amygdala during observation of basic emotions compared with neutral expressions. All five basic emotions dynamically modulated amygdala interactions in a distinctive and category-specific manner, thereby carving a functionally specialized and spatially distributed network for each emotion studied, as displayed in Fig. 2 and further detailed in Tables 1, 2, 3, 4, 5.

The emotion-dependent connectivity pattern of the amygdala can be broadly grouped in five sets, based on their anatomical locations. The first set comprises anterior cortical midline regions, including the dorso-medial prefrontal cortex (dmPFC) and anterior cingulate cortex (ACC). Enhanced amygdala integration with these anterior midline regions characterizes the processing of happy expressions. The second set of inter-regional amygdala connectivity engages posterior area in the medial surface, such as the posterior cingulate cortex (PCC) and the precuneus (PCUN), which are uniquely associated with exposure to angry expressions. The third cluster consists of lateral prefrontal regions, anatomically connected with the amygdala via the uncinate fasciculus^45^, and also extends more posteriorly to premotor, motor and somatosensory areas. We found evidence of greater amygdala connectivity with the dorso-lateral prefrontal cortex (dlPFC) upon perception of sad faces, whereas the other structures in the lateral surface adjacent to the central sulcus are similarly recruited by all emotions and will be discussed below. The fourth set encompasses subcortical areas, such as the thalamus, the olfactory cortex and tubercles (OC), the nucleus accumbens (NAc), ventral pallidum (VP), and the hippocampus (HPC), all forming the signature pattern of amygdala synchrony during exposure to disgust, whereas the brain stem areas, especially in the pons; were unique of anger. The last set involves posterior visual structures, starting from striate cortex (V1) and extending both ventrally, to include the fusiform gyrus (FG) and the superior temporal sulcus (STS) that are anatomically connected with the amygdala via the longitudinal fasciculus^46^, and dorsally, up to the supramarginal gyrus (SMG), which are all distinctive of fear processing.

**Figure 3:**
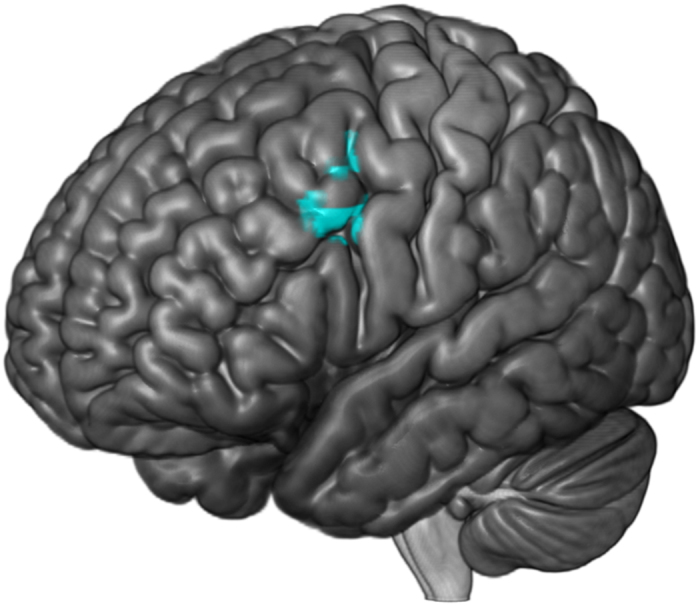
Overlapping regions of significant functional interactions with the amygdala common to all 5 emotions. Results are whole-brain thresholded at *Z* > 2 and cluster corrected at *p* < 0.05.

Finally, we investigated the possible presence of voxels that increase their synchrony with the amygdala in response to all five basic emotions, thus constituting the common core of amygdala functional network shared across emotions. As anticipated, 126 voxels matched this criterion and were localized in the left premotor (precentral gyrus PreCG, BA 6; 76 voxels, MNI coordinates: *X* = −44, *Y* = 2, *X* = 37) and rostral premotor cortex (middle frontal gyrus MFG, BA 8; 48 voxels, MNI coordinates: *X* = −48, *Y* = 18, *X* = 40), known to be involved in motor resonance^47^, action planning and emotional contagion^48^,^49^ (Fig. 3).

## Discussion

Evidence for unique neural fingerprints associated with different emotions remains elusive. In recent years, pattern- or network-based perspectives have gained substantial traction in the quest to characterize emotion-specific brain responses, thereby shifting the focus from single brain structures to the connectivity between multiple regions^2^,^6^,^9^,^10^. While making great strides in overcoming traditional views of one-to-one correspondence between structures and functions, these methods also bear inevitable limitations. One is that they provide a static, instead of dynamic, picture of functional communication between areas. This is particularly noticeable when investigating affective phenomena, owing to the time- and context-dependent role of emotions in flexibly regulating adaptive interactions with our surrounding environment^14^. Therefore, the possibility to better define the neural signature that differentiates univocally each emotion from the others rests also with our ability to understand dynamic modulations of brain connectivity upon processing different emotions; an issue that has come under desultory scrutiny.

On the other hand, there is ample fMRI literature relying on interregional temporal covariance to profile amygdala functional connectivity^31^,^32^,^44^, including task-modulated and dynamic interactions^42^,^43^,^44^. However, amygdala connectional changes are usually compared across broad domains, such as emotion processing, attention, decision-making, or cognitive control, whereas dynamic modulation of functional interactions induced by different instances of basic emotions have not been examined systematically yet. The present study marks an initial attempt to bridge the gap between these two streams of investigation. In fact, we sought to determine if common or segregated patterns of amygdala co-activations exist amidst various emotions, as far as the perception of facial expressions is concerned. Our focus on PPI provides a dynamic perspective on emotion-specific modulation of amygdala connectivity and eschews possible confounds of more traditional methods to assess functional integration that can sample co-occurrence of activation between two or more brain regions but without linking it specifically to task demands or stimulus properties^22^,^23^,^35^. Moreover, we did not apply a priori models nor we restrained on any potential paths of functional amygdala interaction, rather adopting a data-driven and whole-brain approach to increase the robustness of analyses. Our results complement and extend with novel findings the current knowledge on the dynamic signature patterns of neural activity that characterize emotion perception, whilst also contributing a more composite perspective on amygdala functions that goes beyond traditional conceptions of it as fear hub.

First, all five emotional expressions enhanced amygdala activity compared to neutral faces, as reported in the preliminary analysis on fMRI signal change. Our findings are thus in keeping with the results of several neuroimaging investigations showing that the amygdala was comparably active in response to facial expressions of various basic emotions^3^,^30^, and that amygdala voxels contribute to the classification of positive as well as negative emotion categories, such as happiness fear and disgust^6^. The present results also concur with clinical studies reporting that direct simulation of the amygdala can induce either pleasant or unpleasant emotions^50^,^51^. Notably, neglecting the role of dynamic network changes in inter-regional connectivity has led to interpret comparable amygdala responsivity to different emotional expressions as evidence against the existence of unique neural signature for basic emotions^2^.

Second, the emotional expressions modulated amygdala connectivity with a widespread network of cortical and subcortical regions. When considered collectively across emotions, the array of spatially distributed regions interacting with the amygdala includes structures that have all been previously reported in studies on structural or functional amygdala connectivity^7^,^26^,^31^,^32^,^42^,^44^. Moreover, the spatially distributed network of structures interacting with the amygdala is in nice agreement with its connectional pattern, as previously defined by either structural or functional connectome, and parallel clusters and microstructural distinctions within amygdala sub-regions^31^,^32^,^52^. For example, the basolateral cluster is chiefly connected with, and thought to coordinate, lower and higher level visual and associative sensory areas supporting perception of social signals^53^. The central-medial cluster is connected to brain areas implicated in motor behaviour and response preparation, as well as visceral and somatosensory processing^32^. Lastly, the cluster in the superficial nuclei are connected and co-activated with brain areas involved in affiliative and avoidance behaviours or aversion, such as mesolimbic and vmPFC areas, in interoception, olfaction and vegetative processing, such as the NAc, thalamus and brainstem^31^,^32^. Our data, acquired with a 1.5 Tesla scanner, cannot afford the resolution required to discriminate between amygdala sub-nuclei, and future studies will investigate whether changes in amygdala functional connectivity emerge from the preferential recruitment of specific amygdala sub-regions in response to distinct emotions. Nevertheless, the present results suggest the importance of examining the concordance of structural, connectional and functional organization to better characterize the heterogeneity of amygdala functions.

Third, and most noteworthy, for each individual emotion studied the amygdala recruited a starkly distinguishable, but spatially distributed, set of structures to interact with. These changes in amygdala functional connectivity upon exposure to a variety of basic emotions characterize the dynamic signature prototypical of individual emotion processing, and seemingly represent a neural mechanism that serves to implement the distinctive influence that each emotion exerts on perceptual, cognitive, and motor responses. Clearly, the present results are limited to the perception of facial expressions. It remains open to further investigation to establish whether similar changes in amygdala interregional connectivity also occur in response to different classes of emotional stimuli^54^,^55^ or for other aspects of emotion processing beyond perception^56^. It also remains unknown whether gender differences affect amygdala’s dynamic connectional changes, as previously reported for amygdala activity^57^. Happiness and anger were the two emotions inducing higher coupling between amygdala and cortical structures in the medial surface. Nevertheless, there was a sharp distinction along the anterior-posterior axis, with happiness modulating amygdala interactions with left supragenual ACC and bilateral dmPFC, whereas anger enhanced connections with right PCC and PCUN bilaterally. ACC and dmPFC are anatomically interconnected between each other and with the amygdala^58^,^59^, their activity supports both empathic processes^60^ and reward-based decision making^61^, and their response increases when depressed subjects respond positively to pharmacological treatments^62^. Moreover, prior effective connectivity as well as meta-analytic fMRI studies found happiness-related activations in the supracallosal ACC and in the dmPFC, with comparable coordinates to those reported here^4^,^7^. Importantly, in a recent study examining the temporal dynamics of emotion processing, we found that the neuronal generators of EEG responses to happy stimuli were localized in the ACC and dmPFC^14^. This confirms with different methods that have higher temporal, but lower spatial resolution, the time-dependent interaction of these areas contingent upon processing joyful signals.

As for the integration of amygdala activity with posteromedial cortex during anger perception, the PCC and PCUN are reciprocally connected with other limbic and paralimbic structures, consistent with Papez original conceptualization of the PCC as an integral part of the system specialized for emotion processing^63^. These posteromedial areas are involved in detecting and responding to unexpected environmental events with high motivational value that can require a behavioural change^64^,^65^,^66^, as it is appropriate in the case of detecting an angry expression signalling a potentially harm^67^. More specifically, aggressive individuals with poor self-control show greater activity in the PCUN, whose metabolism correlates with negative emotionality^68^. Likewise, PCC and PCUN respond strongly when people are asked to imagine emotionally hurtful events that may induce anger^69^. Finally, increased anger-selective activation has been found in the PCC and PCUN of participants with preclinical Huntington’s disease, of which irritability in an important manifestation^70^. In this context, the anger-specific functional integration of amygdala and brain stem, particularly with the pons, is a quite novel finding, though coherent with human and animal models or aggression. In fact, two prior fMRI studies related anger with activity in the pons. Garfinkel *et al*.^71^ found that anger primes increase systolic blood pressure, speed up reaction times in participants with high anger traits, and enhances activity in the post, which is implicated in the regulation of sympathetic arousal. In another study^72^, angry reactions induced by unfair offers during social interactions activated clusters in the dorso-rostral pons that correspond to the anatomical location of the locus coeruleus^73^, a major source for noradrenalin in the brain, thus critically involved in arousal and stress responses^74^,^75^. Moreover, the raphe nuclei are also located in the region of the pons coherent with the loci co-activated with the amygdala in the present study. These nuclei control forebrain serotonin transmission and their modulation influences aggression^76^,^77^. Admittedly, there is a clear distinction between the perception of angry expressions, as examined in the present study, and the experience of anger, which was not assessed here. Notably, however, the coherence between the structures found here and those reported in prior findings addressing anger experience is in keeping with the view that neural systems related to the perception of emotional signals, on the one hand, and to the expression and experience of the same emotion, on the other, largely overlap^47^,^49^,^78^,^79^,^80^,^81^,^82^. Whether amygdala PPI varies similarly during perception with or without experience of the same emotion awaits future investigation.

Fear was the emotion inducing greater synchrony of amygdala activity with lower- as well as higher-level visual areas, including V1, MOG and areas along the ventral stream, such as the MTG, STS and STG. There is convincing evidence that the amygdala exerts a modulatory influence over visual areas in response to fearful stimuli^83^,^84^ and that this effect is abolished by amygdala lesions, despite visual areas remain functionally and structurally intact^85^. This mechanism enables the amygdala to provide bottom-up attentional modulation toward fearful stimuli, thereby increasing the likelihood that such signals automatically summon attention and reach awareness^79^,^86^. These functional effects are wired in the amygdala back-projections, as direct monosynaptic connections from amygdala neurons reach all cortical stages along the ventral visual system in a topographically-organized manner, including V1^87^. Our results thus extend prior findings showing time- and emotion-dependent dynamic interactions of amygdala and visual areas specific for fear processing.

The perception of disgust enhanced the interaction between the amygdala and cortico-subcortical systems involved in motivating avoidant behaviours, olfaction and memory^31^. These interactions are compatible with the self-boundary function of disgust and its role in prioritizing immediate action generation through revulsion or gag reflex that protect the body from offensive or contaminating entities^88^,^89^. Amygdala enhanced connection with the olfactory (pyriform) cortex is clearly coherent with the ancestral origin of disgust that is anchored on chemical senses, and fits well with behavioural evidence that recognition of disgusted faces is improved by the presentation of an olfactory stimulus irrespective of its emotional valence^90^. Amygdala integration with the NAc and ventral pallidum (VP) finds direct support in rodent studies on disgust, showing that lesions and temporary inactivation of either structures generate intense sensory disgust^91^. The thalamus has been found to respond selectively to disgust in meta-analyses^3^,^10^ or to images of rotten food and mutilation^92^, whereas MVPA found that voxels within the thalamus, especially the ventral anterior nuclei, are particularly accurate in classifying disgust movies clips^6^. Therefore, amygdala connections with various thalamic regions during disgust processing probably serve to selectively amplify and dampen early sensory input to shape environmental perception, whereas enhanced integration with hippocampus (HPC) and parahippocampal cortex (PHC) is functional to store these stimulus-response contingencies in memory. In fact, current evidence seems to support a special role for memory in disgust processing, which possibly originates from its role in conditioned taste aversion. For example, disgusting stimuli enhance episodic memory and seem to have a special salience in memory relative to other equally arousing and negative emotions, such as fear^93^,^94^. Moreover, a recent fMRI study found that disgusting stimuli were the hardest to forget compared to fearful and sad stimuli, and increased amygdala activity along with the hippocampus^95^.

Sadness seems to underlie on tighter and almost exclusive amygdala-dlPFC interactions as compared to the wider network of amygdala co-activations during perception of the other emotions. Although the dlPFC subserves multiple functions and seems to regulate various emotional states^96^,^97^,^98^, recent studies implicate the dlPFC in sadness and depression^99^, especially during cognitive reappraisal and regulation of this emotion^100^. In such tasks, reappraisal minimizes the experience of negative affect after viewing sad stimuli and amygdala responses are dampened, possibly via the inhibitory function of the dlPFC^101^. Likewise, sadness-specific abnormalities and decreased response in the dlPFC have been reported in patients with bipolar disorder and depression, suggesting difficulties in integrating cognitive appraisal with sad experiences in such patients^102^,^103^. Whether the functional connectivity of the amygdala is truly limited to the interaction with dlPFC during sad perception requires further investigation with different stimuli, tasks, and methods of analysis, even though a previous meta-analysis also pointed to a relatively isolated system for sad processing^10^. Moreover, we previously observed with EEG that the temporal course of neural responses to sad images has a much slower and smoother temporal unfolding compared to other emotions^14^. Since the stimuli presented in this study were relatively brief, it may be that the optimal temporal interval to recruit more widespread co-activations with amygdala should extend beyond the time window of the current study, in terms of both stimulus exposure and ISI.

Fourth, and lastly, we investigated areas interacting with the amygdala in all five emotions with respect to neutral faces. All queried emotions enhanced indeed amygdala functional integration with premotor cortices. The coupling of amygdala and motor areas outlines the influence of different emotions in fostering action preparation and planning, as well as motor resonance. In fact, observing emotional stimuli increases motor excitability relative to neutral images^81^,^82^,^104^ and may reflect approach and avoidance preparation^105^, motor mimicry and emotional contagion^48^,^49^,^54^,^106^,^107^,^108^,^109^,^110^. Such functional interaction is consistent with the anatomical evidence indicating white matter connectivity between the amygdala and the motor regions in nonhuman primates^111^ and humans^112^.

In sum, our study demonstrates that anatomically distributed networks of amygdala interactions characterize the dynamic neural signature associated to the perception of individual facial expressions of emotions, and contributes to understand the functions of path of information flow through these interconnected regions. It also provides an initial example to use the ‘task connectome’ derived from PPI analyses as a systematic framework towards understanding the dynamic connectivity underpinning different emotional and cognitive functions or task contexts.

## Methods

### Participants

Seventeen right-handed participants (8 females) with normal or corrected-to-normal vision participated in this study (age M = 21.7, S.D. = 1.4). All subjects were screened for fMRI compatibility had no personal history of neurological or psychiatric illness, drug or alcohol abuse, or current medication. All experimental methods and protocols in the study were performed in accordance with the ethical standards laid down in the 1964 Declaration of Helsinki and approved by the bioethics committee of the University of Turin under the project ‘Neuropsychological bases of emotional and social perception’. All participants provided written informed consent also approved by the same committee.

### Stimuli

The stimuli were taken from Ekman’s series^113^ and displayed facial expressions of anger, disgust, fear, happiness, sadness and emotional neutrality. A total of four different Caucasian actors (2 females) each expressing all 5 emotions and neutral faces were used, for a total of 24 images (6 conditions × 4 actors). All stimuli sustained a visual angle of 8° × 10.5° from a viewing distance of 50 cm from the screen of a 21-in LCD monitor and had a mean luminance of 15 cd/m^2^.

### Procedure

The experiment consisted of an event-related design divided in four runs. In each run, lasting approximately 8 min, all 24 stimuli were presented randomly for a total of 96 trials and 16 repetitions of the same facial expression. Each trial started with a central fixation cross lasting for 1 second against a dark background, followed by stimulus presentation for 1 second. Inter-stimulus interval (ISI) varied randomly within 16–20 seconds time range during which the central cross remained present on the screen. A PC running E-prime controlled stimulus presentation.

Participants lay supine in the scanner with head movements minimized by an adjustable padded head holder. A colour LCD screen projected the stimuli onto a rear projection screen in the bore of the magnet. Participants viewed the screen via an angled mirror system. They were instructed to fixate the central cross during a passive exposure paradigm. This enabled us to record neural activity related to spontaneous emotion processing that was unaffected by spurious factors such as deliberate processing, top-down attentional modulation or action execution and button press. Otherwise, the interpretation of activations in motor and somatosensory cortices, or in the dorsal fronto-parietal attentional network, as predicted from previous studies, had been problematic if they had been concomitant upon these additional task demands. To verify that participants viewed the stimuli, after each run participants were asked whether they had paid attention to the stimuli. All participants responded positively after every run.

### Data Acquisition

Data acquisition was performed on a 1.5 Tesla INTERA™ scanner (Philips Medical Systems) with a SENSE high-field, high-resolution (MRIDC) head coil that was optimized for functional imaging. The functional T2*-weighted images were acquired using echoplanar (EPI) sequences (TR/TE/flip angle = 2000 ms/50 ms/90°; FoV = 256 mm; acquisition matrix was 64 × 64; 19 axial slices slice with 5 mm thickness and 1 mm gap). A total of 240 volumes were acquired covering the whole brain. Two scans were added at the beginning of the functional scanning session and the data discarded to reach a steady-state magnetization before acquiring the experimental data.

In the same session, a set of three-dimensional high-resolution T1-weighted structural images was acquired for each participant using a Fast Field Echo (FFE) sequence (TR/TE/flip angle = 25 ms/7.9/30°; FoV = 256 mm; acquisition matrix was 256 × 256; 160 contiguous 1 mm sagittal slices; isotropic voxel size = 1 × 1 × 1 mm).

### Preprocessing

Functional data were preprocessed using FSL 5.0.9 (www.fmrib.ox.ac.uk/fsl)^114^. Preprocessing steps included skull extraction using Brain Extraction Tool (BET – FSL), bulk and motion correction using rigid transformation (6 degree of freedom), spatial smoothing at 7 mm, high pass temporal filtering set to 128 seconds. Finally, functional and structural data were registered in standard space as follows. Boundary Based Registration approach was used to co-register each functional EPI into the correspondent T1-wighted image, and the same T1-weighted image was then co-registered into MNI152 2 mm^3^ using FMRIB’s Linear Image Registration Tool (FLIRT–FSL). All participants maintained head motion <3 mm for all scans.

The anatomical T1-weighted images were preprocessed using recon-all pipeline (FreeSurfer^115^) to retrieve anatomical parcellation and localize the regions of interest (ROIs) in the amygdale of both hemispheres in each subject. Attention was paid to warrant subject-specific anatomical fit of the ROIs. The FSL transformation matrices were then used to standardize into the MNI152 2 mm^3^ template the FreeSurfer parcellated brain of each participant with the localized amygdala ROIs. After standardization, the ROIs were combined into a single probabilistic map representing the spatial overlapping of the amygdale, as defined anatomically in individual subjects. The ROIs showed excellent spatial overlapping across all subjects and their volumes were consistent with the mean volumes reported in prior MRI and post- mortem studies, indicating a conservative volumetric definition of the amygdale^116^. Lastly, this map was further thresholded to include only voxels common to the ROIs of at least 14 participants (82%).

### fMRI Signal Change in the Amygdala

Before entering fMRI data in the PPI analysis, we first assessed the response in the amygdala to the different facial expressions presented. As previously established for similar cases^117^, we extracted for each participant the time course of BOLD response from amygdala ROIs. The fMRI response was expressed as percentage of BOLD signal change from baseline, defined as the average activity over the whole time course, applying the following formula:


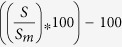


where *S* is the BOLD signal at each time point, and *S*_m_ is the average fMRI response over the whole time course. Lastly, the mean emotion-specific fMRI signal change was computed for each participant and expression independently (i.e., angry, disgusted, fearful, happy, sad, and neutral facial expressions), reflecting the mean peak of stimulus-evoked activity of all voxels in the ROI over a temporal window of 16 sec from stimulus onset.

### PPI Analysis

PPI reveals how activity in a particular brain region (the amygdala in the present case) is differentially correlated (i.e., functionally interacts) with that in other brain regions depending on the experimental conditions (i.e., exposure to different facial expressions)^40^,^41^. Primarily, the mean time series data for the left and right amygdala activity were extracted from the ROIs for each subject and pooled together, thus creating the physiological regressor. To obtain the final PPI results, we implemented the FSL’s 3-level analysis approach. In the first level of the analysis, we designed a general linear model (GLM) approach including the 6 facial expressions of the experimental design (the psychological regressor) convolved with a double-gamma Hemodinamic Response Function (HRF) (zero-centered about the minimum and maximum value of the data) and the (demeaned) amygdala time course. Next, we set the interaction regressor (PPI) between the amygdala time course and the stimulus regressor modelling the 6 expressions^118^. The result of this interaction regressor is the emotion-dependent connectivity of the amygdala with each voxel of the brain. Lastly, we computed a GLM contrast comparing each emotion’s PPI against the PPI for neutral expressions. This enabled us to outline only the unique contribution single emotions by discounting the unspecific effects of face perception.

At the second level, a fixed-effects analysis combined the connectivity data of each subject across all the four runs. At the third level (group level analysis), the obtained contrasts (each emotion vs. neutral) for each subject were modelled for between-subjects variance using mixed effects (FLAME1 option), and the results were cluster-corrected for multiple comparisons at the whole-brain level of *Z* > 2, *p* < 0.05. A preliminary analysis did not reveal any significant gender difference in amygdala connections. Therefore, the data of male and female participants were considered together. The resulting voxel-wise contrast maps were then loaded into MATLAB environment using xjView toolbox (http://www.alivelearn.net/xjview). This enable us to localize anatomically the significant clusters via MNI-based template atlas, to identify active voxels common to all emotions (using the option “common region” in xjView), and to obtain the number of voxels in each these clusters as reported in Tables 1, 2, 3, 4, 5. For display purposes, the activation maps were uploaded in MRIcroGL and the 3D visualization option applied (http://www.mccauslandcenter.sc.edu/mricrogl/home).

## Additional Information

**How to cite this article:** Diano, M. *et al*. Dynamic Changes in Amygdala Psychophysiological Connectivity Reveal Distinct Neural Networks for Facial Expressions of Basic Emotions. *Sci. Rep.* **7**, 45260; doi: 10.1038/srep45260 (2017).

**Publisher's note:** Springer Nature remains neutral with regard to jurisdictional claims in published maps and institutional affiliations.

## Figures and Tables

**Table 1:** Brain areas showing significant functional coupling with the amygdala during perception of angry compared to neutral expressions, as revealed by PPI analysis.

Lobe	Area	Hemisphere	Voxel size	MNI Coordinates		
			(*2* *mm*^3^)	X	Y	Z
Frontal						
	PreCG	L	344	−60	9	23
	IFG	L	206	−53	−4	10
Parietal						
	IPL	L	147	−57	−50	47
	PostCG	L	132	−65	−12	34
	PCUN	L	419	−9	−52	54
		R	279	8	−50	54
	PCC	R	26	11	−44	25
Temporal						
	FG	R	39	32	−38	−13
	STS	R	83	51	−42	11
Occipital						
	V1	R	113	11	−64	16
Brainstem						
	Pons		808	−8	−40	−42

*Abbreviations*: FG, fusiform gyrus; IFG, inferior frontal gyrus; IPL, inferior parietal lobule; PCC, posterior cingulate cortex; PCUN, precuneus; PostCG, postcentral gyrus; PreCG, precentral gyrus; STS, superior temporal solcus; V1, primary visual cortex.

**Table 2:** Brain areas showing significant functional coupling with the amygdala during perception of disgust compared to neutral expressions, as revealed by PPI analysis.

Lobe	Area	Hemisphere	Voxel size	MNI Coordinates		
			(*2* *mm*^3^)	X	Y	Z
Frontal						
	PreCG	L	38	−52	6	31
		R	93	45	−8	50
	dlPFC (MFG)	L	17	−35	42	33
		R	32	44	20	45
Parietal						
	PostCG	R	121	60	−12	43
Temporal						
	TPJ	L	101	−48	−60	20
	ITG	L	194	−52	−49	−13
	HPC	L	30	−18	−8	−15
	FG	L	27	−41	−41	−20
	PHG	L	7	−26	−25	−19
Subcortical						
	POC/NAc/VP	L	58	−22	7	−15
	Thal	L	18	−8	−8	1
		R	9	5	−8	3

*Abbreviations*: PFC, prefrontal cortex; FG, fusiform gyrus; HPC, hippocampus; ITG, inferior temporal gyrus; MFG, middle frontal gyrus; PHG, parahippocampal gyrus; POC/NAc/VP, olfactory cortex, nucleus accumbens, ventral pallidum; PostCG, postcentral gyrus; PreCG, precentral gyrus; Thal, thalamus; TPJ, temporoparietal junction.

**Table 3:** Brain areas showing significant functional coupling with the amygdala during perception of fearful compared to neutral expressions, as revealed by PPI analysis.

Lobe	Area	Hemisphere	Voxel size	MNI Coordinates		
			(*2* *mm*^3^)	X	Y	Z
Frontal						
	dlPFC (MFG)	L	48	−47	12	46
		R	113	44	19	46
	PreCG	L	268	−43	−5	53
		R	290	50	2	48
Parietal						
	PostCG	L	461	−48	−20	54
		R	342	58	−14	43
	SMG	L	153	−62	−30	26
Temporal						
	STS	L	45	−52	−48	9
	STG	L	346	−48	−41	15
	MTG	R	175	45	−58	17
Occipital						
	V1	R	88	16	−64	15
	MOG	R	174	41	−75	6

*Abbreviations*: PFC, prefrontal cortex; MFG, middle frontal gyrus; MOG, middle occipital gyrus; MTG, middle temporal gyrus; PostCG, postcentral gyrus; PreCG, precentral gyrus; SMG, supramarginal gyrus; STG, superior temporal gyrus; STS, superior temporal sulcus; V1, primary visual cortex.

**Table 4:** Brain areas showing significant functional coupling with the amygdala during perception of happy compared to neutral expressions, as revealed by PPI analysis.

Lobe	Area	Hemisphere	Voxel size	MNI Coordinates		
			(*2* *mm3*)	X	Y	Z
Frontal						
	dlPFC (MFG)	L	265	−37	17	37
		R	202	43	21	29
	PreCG	L	280	−37	−3	53
		R	176	42	3	42
	ACC	L	45	−9	35	28
		R	165	6	32	23
	dmPFC (SFG)	L	244	−9	55	38
		R	403	6	57	29
Parietal						
	IPL	R	55	62	−38	41
	PostCG	L	129	−46	−21	53
Temporal						
	MTG	R	141	45	−54	15

*Abbreviations*: ACC, anterior cingulate cortex; dlPFC, dorsolateral prefrontal cortex; dmPFC, dorsomedial prefrontal cortex; IPL, inferior parietal lobule; MFG, middle frontal gyrus; MTG, middle temporal gyrus; PostCG, postcentral gyrus; PreCG, precentral gyrus; STG, superior temporal gyrus.

**Table 5:** Brain areas showing significant functional coupling with the amygdala during perception of sad compared to neutral expressions, as revealed by PPI analysis.

Lobe	Area	Hemisphere	Voxel size	MNI Coordinates		
			(*2* *mm3*)	X	Y	Z
Frontal						
	PreCG	L	449	−49	4	37
	dlPFC	L	279	−46	26	40

*Abbreviations*: dlPFC, dorsolateral prefrontal cortex; PreCG, precentral gyrus.
